# *Trans*-2-enoyl-CoA reductase limits Ca^2+^ accumulation in the endoplasmic reticulum by inhibiting the Ca^2+^ pump SERCA2b

**DOI:** 10.1016/j.jbc.2021.100310

**Published:** 2021-01-19

**Authors:** Yasunori Uchida, Yasunori Yamamoto, Toshiaki Sakisaka

**Affiliations:** Division of Membrane Dynamics, Department of Physiology and Cell Biology, Kobe University School of Medicine, Kobe, Japan

**Keywords:** fatty acid metabolism, endoplasmic reticulum, calcium, calcium ATPase, calcium transport, protein–protein interaction, NFAT transcription factor, DDM, *n*-dodecyl-β-D-maltoside, DMSO, dimethyl sulfoxide, ER, endoplasmic reticulum, FA, fatty acid, FLAG–TER, recombinant TER with an N-terminal FLAG-tag, GPCR, G protein–coupled receptor, GST, glutathione-S-transferase, HBSS, Hank’s balanced salt solution, IP_3_, inositol 1,4,5-triphosphate, KAR, 3-ketoacyl-CoA reductase, LCFA, long-chain fatty acid, NFAT, nuclear factor of activated T cells, PA-SERCA2b, recombinant SERCA2b with an N-terminal PA-tag, pAb, polyclonal antibody, PLN, phospholamban, S1P, sphingosine 1-phosphate, SERCA, sarco(endo)plasmic reticulum Ca^2+^-ATPase, SOCE, store-operated Ca^2+^-entry, SphK, sphingosine kinase, SR, sarcoplasmic reticulum, TER, *Trans*-2-enoyl-CoA reductase, Tg, thapsigargin, VLCFA, very-long-chain fatty acid

## Abstract

The endoplasmic reticulum (ER) contains various enzymes that metabolize fatty acids (FAs). Given that FAs are the components of membranes, FA metabolic enzymes might be associated with regulation of ER membrane functions. However, it remains unclear whether there is the interplay between FA metabolic enzymes and ER membrane proteins. *Trans*-2-enoyl-CoA reductase (TER) is an FA reductase present in the ER membrane and catalyzes the last step in the FA elongation cycle and sphingosine degradation pathway. Here we identify sarco(endo)plasmic reticulum Ca^2+^-ATPase 2b (SERCA2b), an ER Ca^2+^ pump responsible for Ca^2+^ accumulation in the ER, as a TER-binding protein by affinity purification from HEK293 cell lysates. We show that TER directly binds to SERCA2b by *in vitro* assays using recombinant proteins. Thapsigargin, a specific SERCA inhibitor, inhibits this binding. TER binds to SERCA2b through its conserved C-terminal region. TER overexpression suppresses SERCA2b ATPase activity in microsomal membranes of HEK293 cells. Depletion of TER increases Ca^2+^ storage in the ER and accelerates SERCA2b-dependent Ca^2+^ uptake to the ER after ligand-induced Ca^2+^ release. Moreover, depletion of TER reduces the Ca^2+^-dependent nuclear translocation of nuclear factor of activated T cells 4. These results demonstrate that TER is a negative regulator of SERCA2b, implying the direct linkage of FA metabolism and Ca^2+^ accumulation in the ER.

Fatty acids (FAs) are essential constituents of biological membranes, energy storage, and precursor of signaling molecules. Mammalian cells contain FAs with different carbon number, which have both structural and functional diversity. Long-chain FAs (LCFAs) have 11 to 20 carbons, among which C16 and C18 species are the most abundant. Very-long-chain FAs (VLCFAs) have >20 carbons. Although the amount of VLCFAs is generally lower than that of LCFAs, VLCFAs have various functions that are not complemented by LCFAs, such as skin barrier formation, myelin formation and maintenance, retinal function, and spermatogenesis (1, 2, 3, 4). Most of VLCFAs are incorporated into sphingolipids, which are composed of a long-chain base such as sphingosine and an amide-linked acyl chain, to fulfill their functions. Although an LCFA palmitate (C16:0) is synthesized by FA synthase in the cytosol, VLCFAs are synthesized by the FA elongation cycle in the endoplasmic reticulum (ER), which repetitively adds two carbons from malonyl-CoA to acyl-CoA (5, 6). This cycle consists of four reactions: (1) condensation of acyl-CoA with malonyl-CoA to produce 3-ketoacyl-CoA, (2) reduction of 3-ketoacyl-CoA to 3-hydroxyacyl-CoA, (3) dehydration of 3-hydroxyacyl-CoA to *trans*-2-enoyl-CoA, and (4) reduction of *trans*-2-enoyl-CoA to the elongated acyl-CoA. *Trans*-2-enoyl-CoA reductase (TER) is a ubiquitous membrane enzyme that catalyzes the final reduction step of the FA elongation cycle (7). To date, TER is the only known enzyme responsible for this reaction. Consistently, genetic ablation of homologue of TER (*TSC13*) is lethal in yeast because of the lack of the VLCFA synthesis (8).

In addition to the FA elongation, the reductase activity of TER has been shown to be involved in sphingosine 1-phosphate (S1P) metabolism. S1P, which is produced by phosphorylating sphingosine, is a potent lipid mediator that acts as a ligand for five G protein–coupled receptors (GPCR) (S1P_1–5_) (9). The binding of S1P to these receptors controls a variety of physiological processes, such as hematopoietic cell trafficking, immune cell fate decision, and vascular integrity (10). S1P is intracellularly degraded to phosphoethanolamine and *trans*-2-hexadecenal, which are in turn converted to *trans*-2-hexadecenoic acid, followed by formation of *trans*-2-hexadecenoyl-CoA (11, 12). TER has been shown to catalyze reduction of *trans*-2-hexadecenoyl-CoA to palmitoyl-CoA that is then incorporated into other lipids, mostly glycerophospholipids (13). Therefore, TER is involved in both the synthesis of VLCFAs and S1P metabolism, regulating the chemical composition of sphingolipids.

Apart from the reductase activity, TER has been shown to interact with ceramide synthase and Seipin, an ER membrane protein required for lipid droplet biogenesis, thereby associating with ceramide synthesis in the ER membrane and formation of lipid droplets from the ER membrane, respectively (14, 15). However, whether TER associates with other ER processes has not been addressed. Recent genetic studies have shown that mutations in *TECRL*, a muscle-specific paralogue of *TER*, are associated with inherited arrhythmias (16, 17, 18). Interestingly, induced pluripotent cell–derived cardiomyocytes with the *TECRL* mutation exhibit various abnormalities in Ca^2+^ transients upon stimulation, including slower Ca^2+^ uptake to the sarcoplasmic reticulum (SR) (16), although its molecular mechanism remains unclear. It also remains unknown whether TER is involved in Ca^2+^ uptake to the ER in nonmuscle cells.

Ca^2+^ is a ubiquitous signaling molecule that regulates a wide range of cellular processes, such as muscle contraction, neuronal transmission, motility, proliferation, and transcriptional control (19). The ER is the most important intracellular Ca^2+^ store. The Ca^2+^ concentration in the ER is at millimolar levels, whereas the cytosolic Ca^2+^ concentration is at nanomolar levels at rest (19, 20). The ER releases Ca^2+^ into the cytosol through two major classes of Ca^2+^ channels, inositol 1,4,5-triphosphate (IP_3_) receptors (21) and ryanodine receptors (22, 23), in response to various stimuli. The ER then recovers released Ca^2+^ through sarco(endo)plasmic reticulum Ca^2+^-ATPases (SERCAs) that transport Ca^2+^ from the cytosol to the ER lumen with the energy obtained from ATP hydrolysis (24), leading to termination of Ca^2+^ signal. In mammals, three different genes (*ATP2A1-3*) encode SERCA proteins (SERCA1-3). SERCA2, which shows the most widespread expression pattern among three SERCA proteins, has two major splicing isoforms, SERCA2a and SERCA2b. Although SERCA2a is expressed in slow skeletal muscles and cardiac muscles, SERCA2b is expressed ubiquitously and functions as a house-keeping protein (24, 25, 26). Evidence is accumulating that regulation of the SERCA activity is critical to determine the frequency, duration, and strength of Ca^2+^ responses in muscle cells. In cardiac muscle cells, the SERCA2a activity is regulated by phospholamban (PLN), a small transmembrane protein that directly binds to SERCA2a. Although PLN inhibits the SERCA2a activity in resting states, adrenergic stimulation relieves this inhibition. The resultant enhancement of the SERCA2a activity accelerates cardiac relaxation and increases the Ca^2+^ content in the SR, which then strengthens cardiac contraction (27). Although various proteins have been reported to regulate the activity of SERCA in muscle cells (24), the regulatory mechanism of SERCA2b in nonmuscle cells is not completely understood.

In the present study, we identify SERCA2b as a TER-binding protein. We demonstrate that TER overexpression reduces SERCA2b activity. We also show that TER depletion increases the Ca^2+^ content in the ER. In contrast to the *TECRL* mutation, TER depletion accelerates Ca^2+^ uptake to the ER after ligand-induced Ca^2+^ release to the cytosol. These results indicate that TER limits Ca^2+^ accumulation in the ER and reveal a novel regulatory mechanism of SERCA2b in nonmuscle cells.

## Results

### Identification of SERCA2b as a TER-binding protein

**Figure 1:**
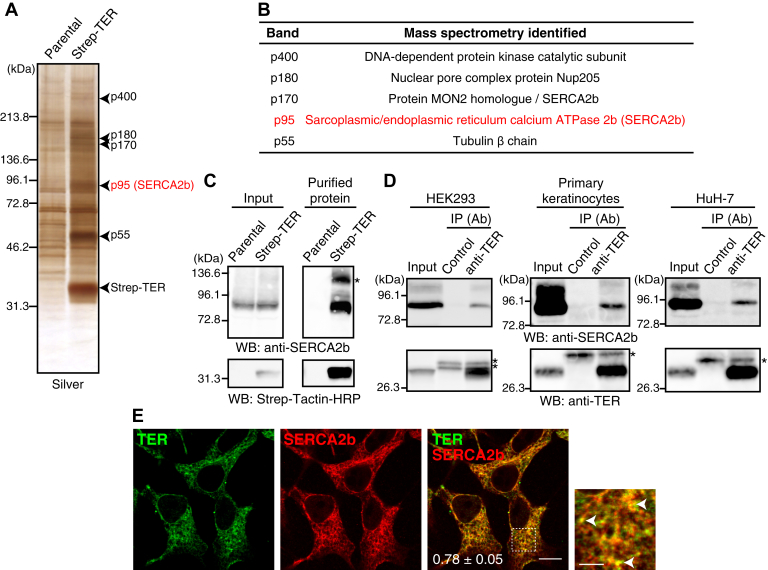
**SERCA2b is a novel binding partner of TER.***A*, purification of Strep-TER–binding proteins. The Triton X-100 extracts of Strep-TER–expressing HEK293 cells or parental cells were applied to Strep-Tactin Sepharose. The bound proteins were eluted with desthiobiotin, and subjected to SDS-PAGE followed by silver staining. The bands indicated by *arrowheads* were cut out and subjected to mass spectrometry analysis. Data are representative of four independent experiments. *B*, the list of Strep-TER–binding proteins. The identified proteins of the bands in panel *A* are shown. *C*, pull-down of endogenous SERCA2b by Strep-TER. The inputs and purified proteins obtained in panel *A* was subjected to Western blotting with anti-SERCA2b mAb and Strep-Tactin-HRP. The *asterisk* (∗) indicates a band corresponding to an SDS-resistant heterodimer of SERCA2b and Strep-TER. Data are representative of three independent experiments. *D*, coimmunoprecipitation of endogenous SERCA2b with endogenous TER. The Triton X-100 extracts of HEK293 cells or the DDM extracts of primary keratinocytes or HuH-7 cells were immunoprecipitated with anti-TER pAb or control Ab and then subjected to Western blotting with anti-TER pAb or anti-SERCA2b mAb. The *asterisks* (∗) indicate nonspecific bands in the immunoprecipitates (IP). Data are representative of three (HEK293) or two (HuH-7) independent experiments. The experiment using primary keratinocytes was performed once. *E*, colocalization of SERCA2b and TER. HEK293 cells were fixed and immunostained with anti-TER pAb and anti-SERCA2b mAb. Data are representative of three independent experiments. The magnified view of the boxed area is shown in the *right* panel. *Arrowheads* indicate the regions where TER and SERCA2b are colocalized. (Scale bar, 10 μm in the merged image and 3 μm in the magnified image). Pearson’s coefficient between TER and SERCA2b is indicated in the merged image (mean ± SD, n = 28 cells). DDM, *n*-dodecyl-β-D-maltoside; pAb, polyclonal antibody; SERCA2b, sarco(endo)plasmic reticulum Ca^2+^-ATPase 2b; TER, *trans*-2-enoyl-CoA reductase.

We first sought to identify ER protein(s) that bind to TER by affinity purification/mass spectrometry. We generated HEK293 clones stably expressing TER with an N-terminal Strep-tag. The Triton X-100 extracts of these cells or parental HEK293 cells were applied to beads conjugated with Strep-Tactin, which binds to the Strep-tag with high affinity and specificity (28). Bound proteins were eluted and subjected to SDS-PAGE followed by silver staining. In addition to Strep-TER, bands at 55, 95, 170, 180, and 400 kDa were specifically detected in the pull-down fraction from Strep-TER–expressing HEK293 cells (Fig. 1*A*). Mass spectrometry analysis identified p55, p95, p170, p180, and p400 as β-tubulin, SERCA2b, protein MON2 homologue/SERCA2b, nuclear pore complex protein Nup205, and DNA-dependent protein kinase catalytic subunit, respectively (Fig. 1*B* and Table S1). Detection of SERCA2b in the p170 band is not consistent with its expected molecular mass (∼100 kDa). Given the identification of the p95 band as SERCA2b, this is probably due to contamination from the p95 band, suggesting the abundance of SERCA2b in the Strep-TER pull-down fraction. The presence of SERCA2b in the Strep-TER pull-down fraction was confirmed by Western blotting with anti-SERCA2b antibody (Fig. 1*C*). In addition to a band with the expected molecular mass (∼100 kDa), the anti-SERCA2b antibody detected a band around 130 kDa in the Strep-TER pull-down fraction. Given that the molecular mass of Strep-TER is about 30 kDa, the 130-kDa band is probably an SDS-resistant heterodimer of SERCA2b and Strep-TER. Because SERCA2b is the only protein localized to the ER in the identified proteins, and expected to be abundant in the Strep-TER pull-down fraction, we characterized SERCA2b as a potential binding partner of TER in this study.

We assessed whether endogenous TER bound to endogenous SERCA2b by coimmunoprecipitation analysis. The Triton X-100 extracts of HEK293 cells were incubated with anti-TER or control antibody. SERCA2b was specifically coimmunoprecipitated with TER (Fig. 1*D*), indicating the binding of endogenous TER to endogenous SERCA2b. We also tested if TER bound to SERCA2b in other cell types. The *n*-dodecyl-β-D-maltoside (DDM) extracts of primary human keratinocytes and HuH-7, a human hepatoma cell line, were subjected to the coimmunoprecipitation analyses. In both cell types, SERCA2b was specifically coimmunoprecipitated with TER (Fig. 1*D*). These results demonstrate that TER binds to SERCA2b not only in HEK293 cells but also in other cell types including primary cells, suggesting the physiological relevance of this interaction. Next, we examined the subcellular localization of TER and SERCA2b. HEK293 cells were immunostained with the anti-TER and the anti-SERCA2b antibodies and examined by confocal fluorescence microscopy. SERCA2b showed a reticular staining pattern throughout the cytoplasm, which is consistent with its well-established ER localization. TER was colocalized with SERCA2b in the reticular structure (Fig. 1*E*). Together, these results indicate that endogenous TER forms a complex with endogenous SERCA2b in the ER membrane.

### Direct and thapsigargin-sensitive binding between TER and SERCA2b

**Figure 2:**
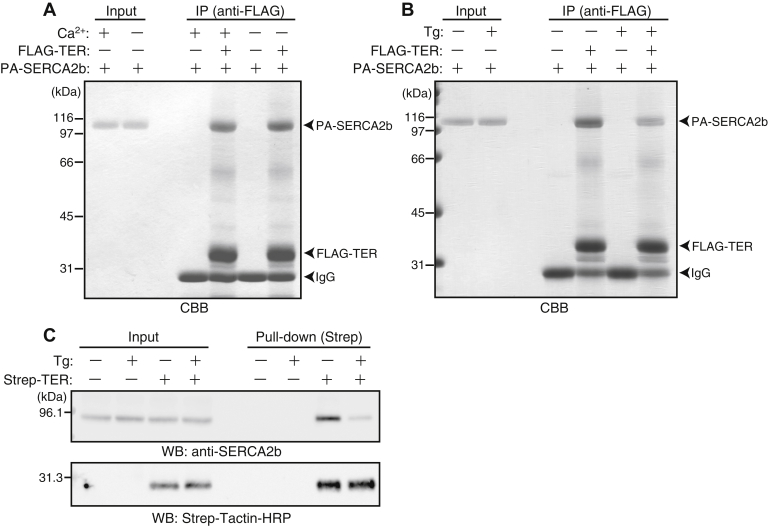
**SERCA2b directly binds to TER in a conformation-dependent manner.***A*, direct binding of SERCA2b to TER in the presence or absence of Ca^2+^. PA-SERCA2b (50 pmol) was incubated with anti-FLAG mAb-conjugated beads with or without immobilized FLAG–TER (125 pmol). The binding reaction was performed in the presence or absence of 100 nM free Ca^2+^. The bound proteins were then eluted with the SDS sample buffer and subjected to SDS-PAGE followed by CBB staining. This experiment was performed once. *B*, inhibition of the direct binding between SERCA2b and TER by Tg. 0.25 μM PA-SERCA2b was treated with or without 2.5 μM Tg, and the binding experiments were then performed as in panel *A* in the presence of 100 nM free Ca^2+^. Data are representative of two independent experiments. *C*, inhibition of the binding between Strep-TER and SERCA2b by Tg. Strep-TER–expressing HEK293 cells or parental cells were treated with or without 1 μM Tg for 90 min. The Triton X-100 extracts of these cells were incubated with Strep-Tactin Sepharose. The bound proteins were then eluted with the SDS sample buffer and subjected to Western blotting with anti-SERCA2b mAb or Strep-Tactin-HRP. Data are representative of four independent experiments. CBB, Coomassie Brilliant Blue; SERCA2b, sarco(endo)plasmic reticulum Ca^2+^-ATPase 2b; TER, *trans*-2-enoyl-CoA reductase; Tg, thapsigargin.

To examine if TER directly bound to SERCA2b, we performed pull-down experiments with purified recombinant proteins. Recombinant TER with an N-terminal FLAG-tag (FLAG–TER) and recombinant SERCA2b with an N-terminal PA-tag (PA-SERCA2b) (29) were expressed in insect cells using baculovirus expression system and purified. PA-SERCA2b was incubated with FLAG–TER immobilized on anti-FLAG antibody-conjugated beads or the anti-FLAG antibody-conjugated beads alone in a buffer containing Ca^2+^. PA-SERCA2b was specifically pulled down with FLAG–TER, indicating that TER directly bound to SERCA2b (Fig. 2*A*). Removing Ca^2+^ from the reaction buffer did not affect the amount of PA-SERCA2b pulled down by FLAG–TER, showing that Ca^2+^ was not essential for the binding of TER to SERCA2b (Fig. 2*A*).

During the pumping cycle, SERCA undergoes conformational changes. The conformations of SERCA are classified into two major states (E1 and E2). The E1 state has high affinity for Ca^2+^ and captures Ca^2+^ in the cytosol, and the E2 state has low affinity for Ca^2+^ and releases Ca^2+^ in the ER lumen (30). Thapsigargin (Tg), a potent inhibitor of SERCA, fixes it to a form analogous to the E2 state, thereby preventing its pumping cycle (31). Pretreatment of purified PA-SERCA2b with Tg reduced the amount of PA-SERCA2b pulled down by FLAG–TER (Fig. 2*B*). We next examined the effect of Tg on the binding of TER to SERCA2b in cells. Strep-TER–expressing HEK293 cells were treated with Tg before cell lysis and subjected to the pull-down experiments. Tg treatment significantly reduced the amount of endogenous SERCA2b pulled down by Strep-TER (Fig. 2*C*). Collectively, these results suggest that SERCA2b in the E2 state has less affinity to TER than that in the E1 state.

### Binding of the TER’s C-terminal region to SERCA2b

**Figure 3:**
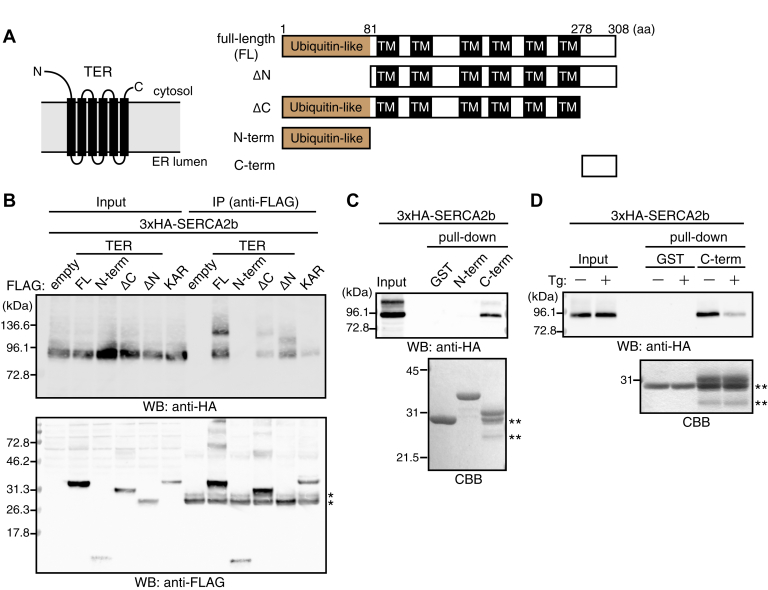
**The C-terminal region of TER is essential for the binding to SERCA2b.***A*, *left*, predicted topology of TER. The topology was based on previous literatures (32, 34). *Right*, domain structure of full-length TER and the deletion mutants used in this study. *B*, coimmunoprecipitation analysis of SERCA2b and TER deletion mutants. 3xHA-SERCA2b and FLAG-empty vector, FLAG–TER FL, FLAG–TER-N-term, FLAG–TER (ΔC or ΔN), or FLAG–KAR were transfected into HEK293 cells. KAR, another essential component of the FA elongation cycle, was included as a negative control. The Triton X-100 extracts of the transfected cells were immunoprecipitated with anti-FLAG mAb and then subjected to Western blotting with anti-HA mAb or anti-FLAG pAb. The *asterisks* (∗) indicate nonspecific bands in the IP. Data are representative of three independent experiments. *C*, binding of the C-terminal region of TER to SERCA2b. 3xHA-SERCA2b was transfected into HEK293 cells, and the Triton X-100 extracts of the transfected cells were incubated with 2.8 nmol of GST, GST-TER-N-term, or GST-TER-C-term immobilized on glutathione Sepharose. The bound proteins were eluted with the SDS sample buffer and subjected to SDS-PAGE followed by Western blotting with anti-HA mAb or CBB staining. Data are representative of two independent experiments. *D*, inhibition of the binding between the C-terminal region of TER and SERCA2b by Tg. HEK293 cells expressing 3xHA-SERCA2b were treated with or without 1 μM Tg for 90 min, and the Triton X-100 extracts of these cells were subjected to the pull-down assay as in panel *C* with 1.9 nmol of GST or GST-TER-C-term immobilized on glutathione Sepharose. This experiment was performed once. The *double asterisks* (∗∗) in panels *C* and *D* indicate the degradation products of GST-TER-C-term. CBB, Coomassie Brilliant Blue; FA, fatty acid; FLAG–TER, recombinant TER with an N-terminal FLAG-tag; GST, glutathione-S-transferase; KAR, 3-ketoacyl-CoA reductase; SERCA2b, sarco(endo)plasmic reticulum Ca^2+^-ATPase 2b; TER, *trans*-2-enoyl-CoA reductase; Tg, thapsigargin; TM, transmembrane region; pAb, polyclonal antibody.

We searched for the region in TER responsible for its binding to SERCA2b. Based on the reported topologies of yeast and *Arabidopsis* orthologues, human TER is predicted to have an N-terminal ubiquitin-like domain in the cytosol, 6 transmembrane helixes, and a short C-terminal cytoplasmic region (32, 33, 34) (Fig. 3*A*, *left*). To test if these cytoplasmic regions were involved in the binding to SERCA2b, we generated truncation constructs lacking either the N- or C-terminal region (ΔN or ΔC) and short fragments containing only one of these regions (N-term or C-term) (Fig. 3*A*, *right*).

FLAG-tagged full-length TER, ΔN, ΔC, and N-term along with 3xHA-tagged SERCA2b were expressed in HEK293 cells, followed by immunoprecipitation with the anti-FLAG antibody. Consistent with the pull-down of SERCA2b by TER (Fig. 2), 3xHA-SERCA2b was significantly coimmunoprecipitated with full-length TER (Fig. 3*B*). To validate the coimmunoprecipitation, we used 3-ketoacyl-CoA reductase (KAR), another enzyme in the FA elongation cycle (7), as a negative control. A faint and negligible level of 3xHA-SERCA2b was coimmunoprecipitated with KAR, validating the specific binding of 3xHA-SERCA2b and full-length TER. 3xHA-SERCA2b was coimmunoprecipitated with ΔN or ΔC much less than with full-length TER (Fig. 3*B*). On the other hand, 3xHA-SERCA2b was not coimmunoprecipitated with N-term. These results suggest that both the N- and C-terminal regions are required for the full binding of TER to SERCA2b and that the N-terminal region by itself does not bind to SERCA2b.

We next investigated if C-term bound to SERCA2b. Because we could not express FLAG-tagged C-term in HEK293 cells, we expressed it as a glutathione-S-transferase (GST)-fusion protein in *Escherichia coli* and performed pull-down experiments. GST, GST-N-term, or GST-C-term was immobilized to glutathione beads, and the beads were incubated with the lysates of HEK293 cells expressing 3xHA-SERCA2b. GST-C-term, but not GST or GST-N-term, pulled down 3xHA-SERCA2b (Fig. 3*C*), indicating that the C-terminal region of TER binds to SERCA2b. These results show that TER directly binds to SERCA2b through its C-terminal region.

Because Tg reduced the binding of full-length TER to SERCA2b (Fig. 2, *B* and *C*), we examined the effects of Tg on the binding of the C-terminal region to SERCA2b. HEK293 cells expressing 3xHA-SERCA2b were treated with Tg before cell lysis, and the lysates of these cells were incubated with GST-C-term immobilized on glutathione beads. Tg treatment reduced the amount of 3xHA-SERCA2b pulled down with GST-C-term (Fig. 3*D*). These results suggest that the C-terminal region prefers SERCA2b in the E1 state to that in the E2 state as full-length TER, validating a role of the C-terminal region as a SERCA2b-interacting region of TER.

We also tested if Tg affected the colocalization of TER and SERCA2b in HEK293 cells by immunofluorescence microscopy. TER and SERCA2b were still colocalized in Tg-treated cells (Fig. S1). Because TER and SERCA2b are ER-resident proteins, they are probably present in the ER membrane even after they are dissociated. Therefore, it might be difficult to detect changes in the colocalization of these proteins with Tg treatment by immunofluorescence microscopy.

### Suppression of SERCA2b activity by TER

**Figure 4:**
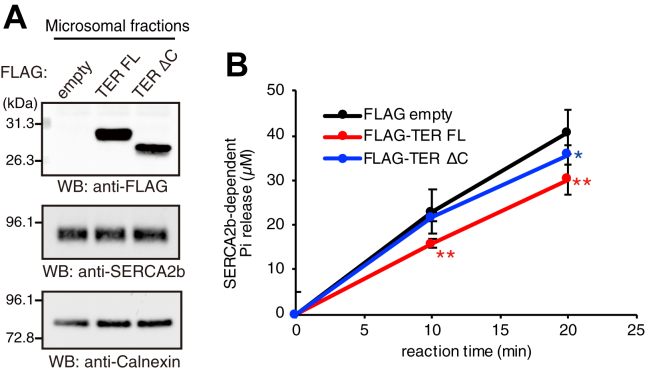
**Reduction of the SERCA2b ATPase activity by TER overexpression.***A*, protein levels of FLAG-tagged TER and SERCA2b in microsomal fractions. FLAG-empty vector, FLAG–TER FL, or ΔC was transfected into HEK293 cells. The microsomal fractions were then prepared by differential centrifugation. The samples were subjected to Western blotting with anti-FLAG mAb, anti-SERCA2b mAb, or anti-Calnexin mAb. Data are representative of four independent experiments. *B*, SERCA2b ATPase assay. The SERCA2b-dependent release of inorganic phosphate (P_i_) from ATP was measured with 30 μg of the microsomal proteins. Data represent the mean ± SD from four independent experiments. ∗*p* < 0.05 and ∗∗*p* < 0.01 *versus* FLAG-empty vector, two-tailed *t*-test. SERCA2b, sarco(endo)plasmic reticulum Ca^2+^-ATPase 2b; TER, *trans*-2-enoyl-CoA reductase.

Tg inhibits the binding of SERCA to PLN and sarcolipin, both of which regulate SERCA activity in muscle cells (35). Therefore, our finding that Tg inhibits the binding of TER to SERCA2b (Fig. 2, *B* and *C*) raised the possibility that TER would modulate SERCA2b activity. To test this possibility, we examined the effect of TER overexpression on endogenous SERCA2b ATPase activity. FLAG–TER was transiently expressed in HEK293 cells, and a microsomal fraction was prepared by differential centrifugation. The endogenous SERCA2b ATPase activity in the microsomal membranes was measured with a spectrophotomeric assay (36). FLAG–TER (full-length or ΔC) expression did not alter SERCA2b protein levels in the microsomal fractions (Fig. 4*A*). However, FLAG–TER (full-length) expression significantly reduced SERCA2b ATPase activity compared with empty vector control. The expression of the TER mutant with little SERCA2b binding (ΔC) weakly reduced SERCA2b activity (Fig. 4*B*). These results demonstrate that TER expression reduces SERCA2b activity through its binding to SERCA2b.

**Figure 5:**
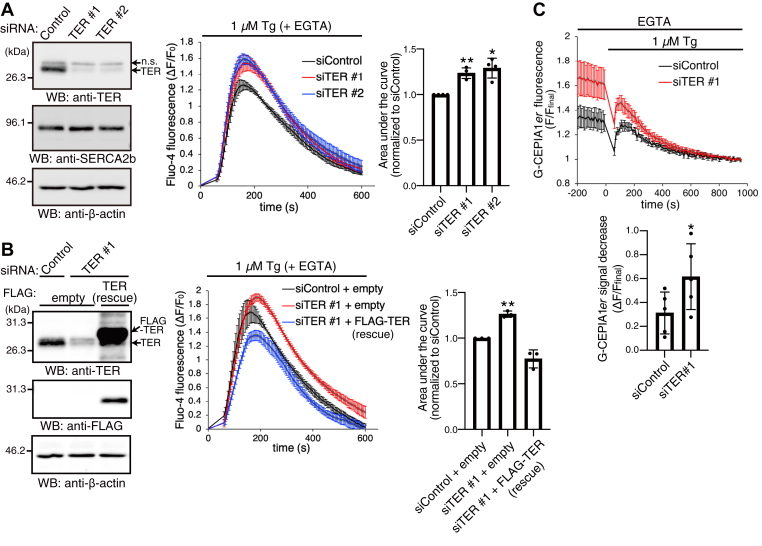
**TER limits the Ca**^**2+**^**content in the ER lumen.***A*, increase of the ER Ca^2+^ content by TER depletion. siControl or siTER (#1 or #2) was transfected into HEK293 cells. After 72 h, the cells were subjected to the following experiments. The whole-cell lysates were subjected to Western blotting with anti-TER pAb, anti-SERCA2b mAb, or anti-β-actin mAb. The cells were loaded with a cytosolic Ca^2+^ indicator dye, Fluo-4, and then incubated in a Ca^2+^-free buffer supplemented with 0.5 mM EGTA. The Ca^2+^ in the ER lumen was released by adding 1 μM Tg (time = 0 s), and the Fluo-4 fluorescence was measured every 5 s for 10 min. The changes in fluorescence values relative to baseline (ΔF/F_0_) were plotted as a function of time. The areas under the curves, which were normalized to the siControl values, were shown in a bar graph. Data represent the mean ± SEM in the traces, or the mean ± SD with data points plotted in the bar graph, from four independent experiments. *B*, rescue of the siTER #1 effects by reconstituting TER expression. The indicated siRNA and plasmid were transfected into HEK293 cells. After 72 h, the cells were subjected to the subsequent experiments as in panel *A*. The whole-cell lysates were subjected to Western blotting with anti-TER pAb, anti-FLAG mAb, or anti-β-actin mAb. Data represent the mean ± SEM in the traces, or the mean ± SD with data points plotted in the bar graph, from three independent experiments. *A* and *B*, ∗*p* < 0.05 and ∗∗*p* < 0.01 *versus* the theoretical control value of 1.0, two-tailed one-sample *t*-test. *C*, direct measurement of the ER Ca^2+^ levels with a genetically encoded Ca^2+^ indicator. At 48 h after siRNA transfection, HEK293 cells were transfected with the indicator (G-CEPIA1*er*) and further incubated for 24 h. The cells were then incubated in a Ca^2+^-free buffer supplemented with 0.5 mM EGTA and treated with 1 μM Tg (time = 0 s). The G-CEPIA1*er* fluorescence was measured every 10 s for 15 min. The fluorescence values relative to the final fluorescence values (F/F_final_) were plotted as a function of time. The relative decrease of the G-CEPIA1*er* fluorescence values (ΔF/F_final_) was shown in a bar graph. Data represent the mean ± SEM in the traces, or the mean ± SD with data points plotted in the bar graph, from five independent experiments. ∗*p* < 0.05 *versus* siControl, two-tailed paired *t*-test. ER, endoplasmic reticulum; n.s., nonspecific bands; SERCA2b, sarco(endo)plasmic reticulum Ca^2+^-ATPase 2b; TER, *trans*-2-enoyl-CoA reductase; Tg, thapsigargin.

SERCA2b ATPase activity is required for uptake of Ca^2+^ from the cytosol to the ER lumen. We therefore hypothesized that TER would limit the Ca^2+^ content in the ER lumen by suppressing SERCA2b activity. To address this, we measured the ER Ca^2+^ content in cells depleted of TER. Two independent siRNAs were used to deplete TER, which was confirmed by Western blotting with the anti-TER antibody (Fig. 5*A*). SERCA2b protein levels were grossly normal in TER-depleted cells when compared with control cells. To monitor the ER Ca^2+^ content, we treated cells with Tg in the absence of extracellular Ca^2+^. This treatment causes the passive release of Ca^2+^ from the ER lumen to the cytosol. This release can be measured as an increase in the fluorescence value of a cytosolic Ca^2+^ indicator dye, Fluo-4 (37). The increase of Fluo-4 fluorescence after Tg addition was larger in TER-depleted cells than in control cells (Fig. 5*A*). To estimate the total amount of Ca^2+^ released to the cytosol, we calculated the area under the curve of each Ca^2+^ trace. The areas under the curves were significantly increased by TER depletion (Fig. 5*A*), suggesting that TER depletion increased the ER Ca^2+^ content.

We next examined if these abnormalities in TER-depleted cells was rescued by reconstituting TER expression. Transfection of an siTER #1-resistant TER construct [FLAG–TER (rescue)] with siTER #1 clearly restored TER expression (Fig. 5*B*). Consistent with the results in cells without vector transfection (Fig. 5*A*), the increase of Fluo-4 fluorescence after Tg addition and the areas under the curves were larger in cells transfected with siTER #1 and empty vector than in cells transfected with siControl and empty vector. In contrast, they were not significantly changed in cells transfected with siTER #1 and FLAG–TER (rescue) compared with those in the control cells (Fig. 5*B*). These results indicate that the increase of the ER Ca^2+^ content by siTER #1 transfection is rescued by reconstituting TER expression, eliminating the possibility that the effect of siTER #1 is an off-target effect.

To further corroborate that TER depletion increases the ER Ca^2+^ levels, we directly measured the ER Ca^2+^ levels with a genetically encoded Ca^2+^ indicator called G-CEPIA1*er*, which is an ER luminal protein that enhances its green fluorescence upon Ca^2+^ binding (20). HEK293 cells were transiently transfected with G-CEPIA1*er* after siRNA transfection. To determine the ER Ca^2+^ levels, we depleted the ER Ca^2+^ with Tg in the absence of extracellular Ca^2+^ and measured the reduction of G-CEPIA1*er* fluorescence. This reduction (ΔF) was normalized to the final fluorescence value after the depletion of the ER Ca^2+^ with Tg (F_final_). This value was supposed to be the minimum fluorescence value of G-CEPIA1*er* without Ca^2+^ binding, therefore could be used as an internal control of the G-CEPIA1*er* transfection efficiency. The ΔF/F_final_ values were significantly larger in TER-depleted cells than in control cells (Fig. 5*C*), indicating that the ER Ca^2+^ levels in TER-depleted cells were higher than those in control cells. Taken together, these results demonstrate that TER limits the Ca^2+^ content in the ER lumen by suppressing SERCA2b activity.

### Role of TER in ligand-induced cytosolic Ca^2+^ responses and NFAT nuclear translocation

The SERCA2b-mediated Ca^2+^ uptake to the ER is one of the primary mechanisms to terminate cytosolic Ca^2+^ responses elicited by ligands of cell surface receptors in nonexcitable cells (38). Because TER limits SERCA2b activity, we reasoned that TER extends the duration of cytosolic Ca^2+^ responses. Cytosolic Ca^2+^ responses were typically elicited by GPCRs (primary G_q/11_ subtypes) that trigger phospholipase C–mediated IP_3_ production, which in turn releases Ca^2+^ from the ER *via* IP_3_ receptors (19). HEK293 cells endogenously express the P2Y receptors coupled to phospholipase C activation (39). We thus treated HEK293 cells with extracellular ATP, a ligand for the P2Y receptors, and examined cytosolic Ca^2+^ responses with Fluo-4.

**Figure 6:**
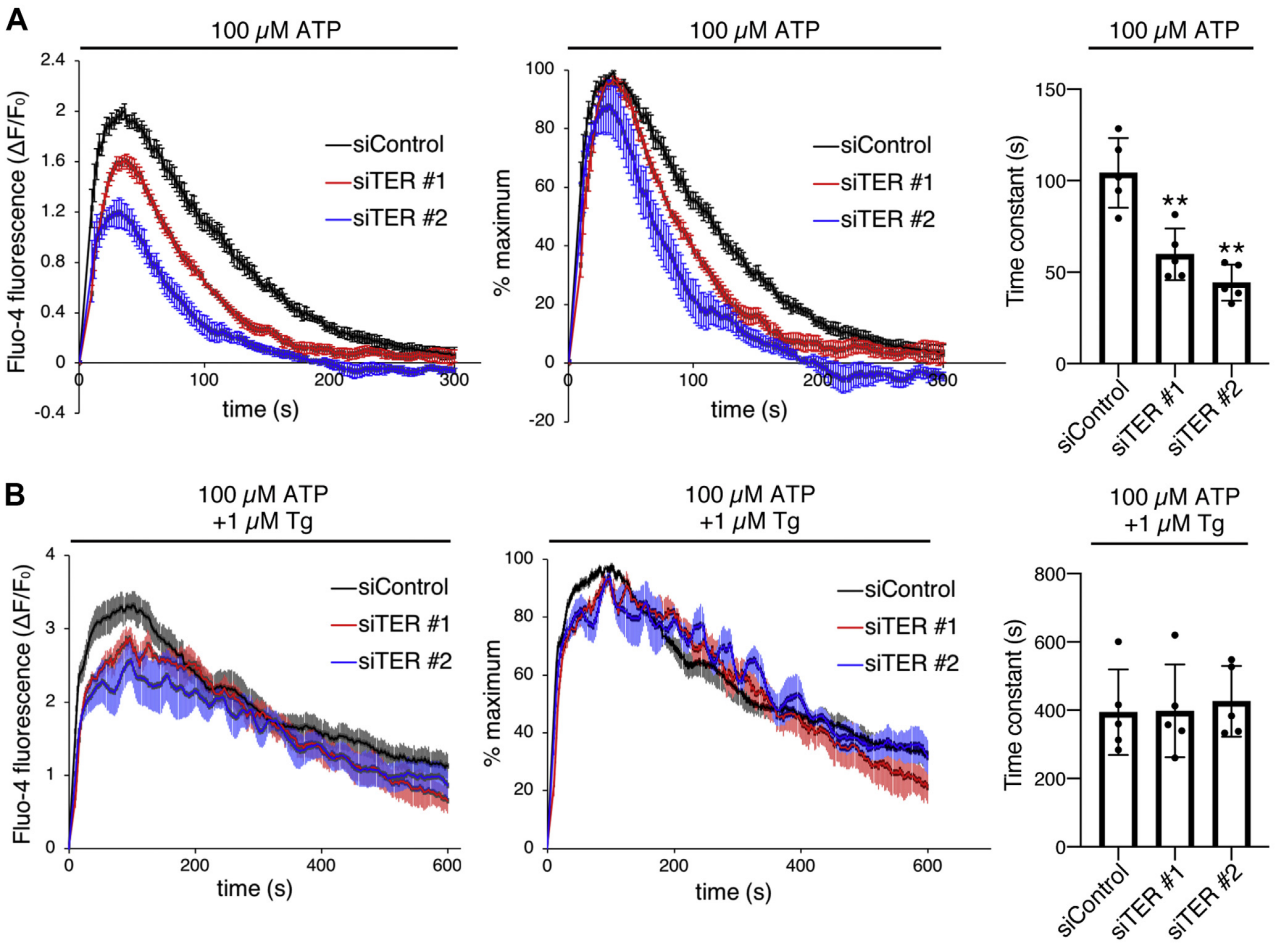
**TER depletion shortens the duration of cytosolic Ca**^**2+**^**responses in a SERCA2b-dependent manner.***A*, Ca^2+^ responses elicited by ATP. siControl or siTER (#1 or #2) was transfected into HEK293 cells. After 48 h, the cells were loaded with Fluo-4 and then incubated in Ca^2+^-containing buffer. After 30 min, 100 μM ATP was added (time = 0 s), and the Fluo-4 fluorescence was measured every 2 s for 5 min. The changes in fluorescence values relative to baseline (ΔF/F_0_) were plotted as a function of time in the *left* graph. To visualize the kinetics of the Ca^2+^ responses, the ΔF/F_0_ values were normalized to the maximum value in each trace and are shown in the *middle* graph. Decay time constants of the Ca^2+^ traces, which were determined by single exponential curve fitting, are shown in a bar graph in the *right*. Data represent the mean ± SEM in the traces, or the mean ± SD with data points plotted in the bar graph, from five independent experiments. ∗∗*p* < 0.01 *versus* siControl, two-tailed *t*-test. *B*, Ca^2+^ responses elicited by ATP in the presence of Tg. Cells were treated as in panel *A*, except that ATP was added with 1 μM Tg and the fluorescence was measured for 10 min. Data represent the mean ± SEM in the traces, or the mean ± SD with data points plotted in the bar graph, from five independent experiments. SERCA2b, sarco(endo)plasmic reticulum Ca^2+^-ATPase 2b; TER, *trans*-2-enoyl-CoA reductase; Tg, thapsigargin.

In control cells, ATP application caused a rapid increase of Fluo-4 fluorescence, indicating a rise of cytosolic Ca^2+^ concentration. This increase peaked at around 30 s, and the Fluo-4 signals gradually decreased to the baseline within 300 s. In TER-depleted cells, the Ca^2+^ responses decreased faster and terminated earlier than in control cells (Fig. 6*A*). The faster decrease of Ca^2+^ responses was more obvious when the Fluo-4 signals were normalized to the maximum value of each trace (Fig. 6*A*). To quantify the decay kinetics, we fitted our data to single exponential curves and calculated decay time constants. The decay time constants were significantly smaller in TER-depleted cells, indicating the shorter duration of Ca^2+^ responses (Fig. 6*A*). To examine if these observations in TER-depleted cells were due to increased SERCA2b activity, we examined the Ca^2+^ responses in the presence of Tg (Fig. 6*B*). In this condition, Ca^2+^ response curves in TER-depleted cells were similar to those of control cells. Furthermore, decay time constants were not decreased by TER depletion. Together, these results indicate that TER depletion shortens the duration of cytosolic Ca^2+^ responses by increasing SERCA2b activity, suggesting a role of TER in sustaining ligand-induced Ca^2+^ signaling.

Notably, the maximum amplitudes of cytosolic Ca^2+^ responses after ATP application were somewhat lower in TER-depleted cells than in control cells (Fig. 6*A*) despite the increased ER Ca^2+^ levels in TER-depleted cells (Fig. 5, *A* and *C*). The purinoceptor experiments were conducted in the presence of extracellular Ca^2+^. On the other hand, the ER Ca^2+^ levels were examined in the absence of extracellular Ca^2+^. To test if the presence of extracellular Ca^2+^ contributes to this discrepancy, we conducted the purinoceptor experiments in the absence of extracellular Ca^2+^. In this condition, the maximum amplitudes of the Ca^2+^ responses were much lower in TER-depleted cells than in control cells (Fig. S2*A*), indicating that TER depletion attenuated the Ca^2+^ responses regardless of the presence of extracellular Ca^2+^. Importantly, in the presence of Tg, TER depletion did not reduce the maximum amplitudes of the Ca^2+^ responses (Fig. S2*B*). The overall Ca^2+^ responses after ATP application with Tg were slightly larger in TER-depleted cells than in control cells, consistent with the increased ER Ca^2+^ levels in TER-depleted cells. These results suggest that the increased SERCA2b activity is involved in the attenuation of the ATP-triggered Ca^2+^ responses in TER-depleted cells.

Finally, we sought physiological function of the Ca^2+^ regulation by TER. One of the major functions of cytosolic Ca^2+^ signaling is transcriptional control through Ca^2+^-dependent transcription factors (19). Of particular interest, sustained rise of cytosolic Ca^2+^ triggers nuclear translocation of the nuclear factor of activated T cells (NFAT) family of transcription factors (40, 41). Given that TER sustained cytosolic Ca^2+^ responses in the presence of extracellular Ca^2+^ (Fig. 6*A*), we reasoned that TER would enhance the nuclear translocation of NFAT. Among four NFAT isoforms (NFAT1-4), NFAT4 has been shown to robustly translocate to the nucleus after GPCR stimulation (42). We thus tested if TER depletion reduced the nuclear translocation of NFAT4 after ATP application.

**Figure 7:**
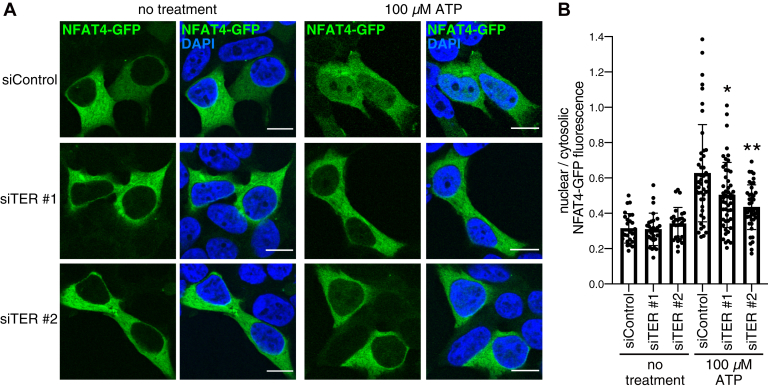
**TER depletion reduces the nuclear translocation of NFAT4-GFP.***A*, nuclear translocation of NFAT4-GFP after ATP application. At 48 h after siRNA transfection, HEK293 cells were transfected with NFAT4-GFP and further incubated for 24 h. The cells were then incubated in a serum-free media for 4 h and treated with 100 μM ATP for 5 min. After fixation, the cells were stained for nuclei with DAPI. Data are representative of two independent experiments. (Scale bar, 10 μm). *B*, quantification of the results in panel *A*. The ratio of nuclear NFAT4-GFP fluorescence intensity to cytosolic NFAT4-GFP fluorescence intensity was calculated and plotted with the mean ± SD in a bar graph. n ≥ 27 cells (no treatment) or ≥41 cells (100 μM ATP) from two independent experiments. The data were analyzed by two-way ANOVA (100-μM ATP: F = 69.33, *p* < 0.0001; the siRNAs: F = 4.288, *p* = 0.0149; interaction: F = 6.786, *p* = 0.0014). ∗*p* < 0.01 and ∗∗*p* < 0.001 *versus* siControl (100 μM ATP), Tukey’s post hoc test. DAPI, 4',6-diamidine-2'-phenylindole; NFAT4, nuclear factor of activated T cells 4; TER, *trans*-2-enoyl-CoA reductase.

To monitor the subcellular localization of NFAT4, we used the N-terminal Ca^2+^-responsive region of NFAT4 tagged with a C-terminal GFP-tag (NFAT4-GFP), which has been frequently used for this purpose (42, 43, 44). HEK293 cells were transfected with NFAT4-GFP after siRNA transfection. After 5 min of ATP treatment, cells were fixed and examined by confocal microscopy. As expected, some portion of NFAT4-GFP was translocated to the nucleus by ATP treatment in control cells. The nuclear translocation of NFAT4-GFP was apparently reduced in TER-depleted cells (Fig. 7*A*). To quantify the results, we calculated the ratio of nuclear NFAT4-GFP fluorescence intensity to cytosolic NFAT4-GFP fluorescence intensity in each cell. The obtained nuclear-to-cytosolic ratio of NFAT4-GFP fluorescence intensity was significantly lower in TER-depleted cells than in control cells (Fig. 7*B*). These results indicate that TER enhances the Ca^2+^-dependent nuclear translocation of NFAT4, implicating that TER plays a role in transcriptional activation by NFAT4.

## Discussion

TER is an FA reductase in the ER membrane, which plays an essential role in the FA elongation cycle and S1P metabolism. In this study, we biochemically purified and identified TER-binding proteins and showed that TER directly binds to an ER Ca^2+^ pump SERCA2b. We further demonstrated that TER is a negative regulator of SERCA2b activity and implicated a role of TER in sustaining cellular Ca^2+^ signaling. Overall, our results pointed out a previously unrecognized function of TER in limiting Ca^2+^ accumulation in the ER.

**Figure 8:**
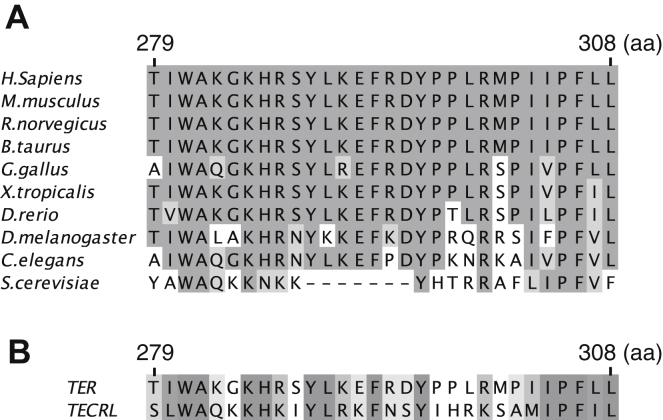
**Conservation of the C-terminal cytoplasmic region in TER homologues.***A*, sequence alignment of TER orthologues from various species in their C-terminal regions. *B*, sequence alignment of human TER and human TECRL in their C-terminal regions. Amino acid numbers in human TER are shown above the alignment. Identical and similar residues are shaded in *dark gray* and *light gray*, respectively. TECRL, *trans*-enoyl-CoA reductase-like; TER, *trans*-2-enoyl-CoA reductase.

The binding to SERCA2b is mediated by the TER’s C-terminal cytoplasmic region (279–308aa). This region is conserved in various organisms, suggesting that the inhibition of the SERCA activity is an evolutionally conserved role of TER (Fig. 8*A*). Although most species contain SERCA-like Ca^2+^ pumps in their genome, *Saccharomyces cerevisiae* lacks such pumps (24). Interestingly, the TER orthologue in *S. cerevisiae* lacks several amino acid residues in the C-terminal region, which are mostly conserved in other species, raising the possibility that these amino acids may be engaged in the binding of TER to SERCA.

Our binding experiments show that TER binds to SERCA2b in the presence or absence of Ca^2+^ (Fig. 2*A*) and that Tg inhibits this binding (Fig. 2, *B* and *C*). SERCA2b in the Ca^2+^-free buffer was probably in the E1 state because the pH of the buffer was 7.0, at which SERCA1a has been shown to be predominantly in the E1 state even in the absence of Ca^2+^ (45). Together, these results suggest that TER preferentially binds to SERCA2b in the E1 state regardless of Ca^2+^ binding compared with that in the E2 state. Structural studies of SERCA1a have shown that the cytoplasmic domains are open in the E1 state, but they are closely associated each other, creating a compact headpiece in the E2 state (45, 46, 47, 48). Such structural rearrangements likely occur in SERCA2b, given that the E1 conformation of SERCA2b is quite similar to that of SERCA1a (36). Therefore, the cytoplasmic regions specifically exposed in the open E1 state are probably involved in the binding of SERCA2b to TER. In the E1 state, SERCA2b binds to Ca^2+^ and then receives ATP from the cytosol, followed by the phosphorylation of its own aspartate residue. These processes are essential for the subsequent transport of Ca^2+^ to the ER lumen (47). Given TER likely binds to SERCA2b both in the E1 state and in the Ca^2+^-bound E1 state, TER may hinder the ATP binding and/or the phosphoryl transfer to SERCA2b in the Ca^2+^-bound E1 state, thereby reducing the catalytic rate of SERCA2b.

TER binds to SERCA2b directly (Fig. 2*A*) and reduces SERCA2b activity through its binding to SERCA2b (Fig. 4*B*), suggesting that the direct binding of TER inhibits SERCA2b. On the other hand, we cannot eliminate the possibility that TER inhibits SERCA2b indirectly through synthesizing acyl-CoAs. Of note, phosphatidylcholine with saturated FAs has been shown to inhibit SERCA2b (49). TER produces various saturated acyl-CoAs, which are then incorporated into membrane lipids. It is thus possible that TER increases saturated FAs within membrane lipids in the local vicinity of SERCA2b, which hampers the catalytic reaction of SERCA2b. Further investigations will be needed to clarify the molecular mechanism by which TER inhibits SERCA2b.

Whether the binding of TER to SERCA2b is constitutive or regulated is an open question. One possibility is that it is regulated by post-translational modifications of TER or SERCA2b. Notably, proteomic studies show that a serine residue (Ser58) in the N-terminal cytoplasmic region of TER is phosphorylated (50, 51). Because the N-terminal region is required for the full binding of TER to SERCA2b (Fig. 3*B*), it is possible that this putative Ser58 phosphorylation affects the binding of TER to SERCA2b. Thus, protein kinases or phosphatases targeting Ser58 may regulate the binding of TER to SERCA2b.

It is well established that the IP_3_ receptor–mediated Ca^2+^ release increases the cytosolic Ca^2+^ and reduces the ER Ca^2+^. Reduction of the ER Ca^2+^ then activates the store-operated Ca^2+^-entry (SOCE), which incorporates Ca^2+^ from the extracellular space to further increase the cytosolic Ca^2+^ (52). SERCA2b then refills the ER with Ca^2+^, leading to termination of Ca^2+^ signaling. TER depletion shortens ATP-induced cytosolic Ca^2+^ responses in the presence of extracellular Ca^2+^ (Fig. 6*A*). Therefore, one simple explanation for this is that TER would limit the SERCA2b activity after SOCE, thereby sustaining the cytosolic Ca^2+^ responses. On the other hand, there exists another possibility that TER would limit the SERCA2b activity during SOCE, which in turn strengthens the reduction of ER Ca^2+^, leading to enhanced activation of the SOCE pathway. Given that SOCE is essential for the NFAT nuclear translocation (44, 52), the enhancement of the NFAT4 nuclear translocation by TER (Fig. 7) also supports a role of TER in upregulating SOCE. Although an earlier study has shown that SOCE is not significantly activated downstream of ATP in HEK293 cells (53), the duration of the Ca^2+^ responses after ATP application was longer in the presence of extracellular Ca^2+^ (∼300 s) than in the absence of extracellular Ca^2+^ (∼100 s) in the present study (Fig. 6*A* and Fig. S2*A*). We therefore assume that SOCE contributes, at least in our experimental setup, to the ATP-triggered Ca^2+^ responses. The ATP-induced rise of cytosolic Ca^2+^ has been shown to induce secretion of other signaling molecules, such as neurotransmitters, cytokines, and hormones (54). Because SERCA2b and TER are expressed ubiquitously, TER likely augments ATP-induced secretion in various cell types by sustaining Ca^2+^ responses. Our results also suggest that TER is a positive regulator of NFAT4 (Fig. 7). Because NFATs govern a wide range of physiological processes, including immune cell activation, tissue development, and cancer progression (41, 55), the present study raises the intriguing possibility that TER will be involved in these processes by enhancing NFAT activity.

TER depletion reduces the maximum amplitudes of Ca^2+^ responses after ATP application, which is rescued by inhibiting SERCA2b activity by Tg (Fig. 6 and Fig. S2). One possible mechanism underlying these results is that SERCA2b takes up more Ca^2+^ from the cytosol to the ER during the opening of IP_3_ receptors in TER-depleted cells than in control cells, thereby reducing the size of the net efflux of Ca^2+^ to the cytosol after ATP application. However, it is also possible that TER depletion attenuates the Ca^2+^ responses by other mechanisms, such as inhibiting purinoceptor activation or IP_3_ receptor opening. Further studies are required to clarify how TER shapes cytosolic Ca^2+^ response after GPCR activation.

The increase of cytosolic Ca^2+^ concentration induces various changes in lipid metabolism (56). Of particular interest, the activity of sphingosine kinase (SphK), an enzyme synthesizing S1P from sphingosine, is stimulated by increasing cytosolic Ca^2+^ concentration (57). This observation suggests that TER may enhance the SphK activity by sustaining cytosolic Ca^2+^ responses. Indeed, TER knockdown in HeLa cells has been shown to reduce the SphK activity (13). The enzymatic activity of TER is also required for the metabolism of S1P to palmitoyl-CoA. Therefore, we speculate that TER induces the production of S1P for this metabolic pathway. Because this pathway is essential for the degradation of sphingolipids, the inhibition of the SERCA2b activity by TER may be particularly important for the regulation of cellular sphingolipid levels.

The mutation in the *TECRL* gene leads to abnormal Ca^2+^ transients in cardiomyocytes. This mutation, however, reduces the Ca^2+^ uptake to the SR, suggesting that TECRL is a positive regulator of the SERCA2a activity (16). This is not consistent with our present finding that TER limits the SERCA2b activity. We postulate possible mechanisms underlying these observations. TECRL may bind to SERCA2a and increase its activity. Although the C-terminal amino acid residues of TECRL are similar to those of TER, there are some distinct residues (Fig. 8*B*), implying that TECRL affects SERCA2a differently from TER. Also, because TER is coexpressed with TECRL in muscle tissues (16), TECRL may compete with TER for the SERCA2a binding, thereby suppressing the negative effect of TER on the SERCA2a activity. Addressing these possibilities will help understand the molecular basis of the disease caused by the *TECRL* mutations.

The point mutation (P182L) of the *TER* gene was identified in patients of nonsyndromic mental retardation (58). This mutation reduces the activity and stability of the TER protein, which leads to the slight decrease of VLCFA-containing sphingolipids (34). Because TER depletion accelerated the termination of cytosolic Ca^2+^ responses, reduced TER protein expression in the patients with this mutation probably leads to similar Ca^2+^ signaling defects. Ca^2+^ is a central regulator of neuronal function. For instance, Ca^2+^ elevation in synapses initiates the biochemical cascade that leads to long-term potentiation of synaptic strength, which has an important role in learning and memory (59). Interestingly, TER was originally cloned as a synaptic glycoprotein, implying the abundance of TER in synapses (60). Therefore, it is tempting to speculate that TER enhances synaptic functions by sustaining Ca^2+^ responses.

## Experimental procedures

### Plasmids

The plasmid containing a cDNA encoding human TER (ID: FXC11442), human KAR (ID: FXC08601), or human SERCA2b (ID: ORK11825) was purchased from Kazusa DNA Research Institute. A cDNA encoding the N-terminal Ca^2+^-responsive region (1–407aa) of human NFAT4 (43) was amplified by PCR from human brain cDNA library (TAKARA). For stable transfection in mammalian cells, the cDNA encoding TER was subcloned into the pCAGIpuro vector with an N-terminal Strep-tag. For transient transfection in mammalian cells, the cDNA encoding TER or KAR was subcloned to pCMV vector with an N-terminal FLAG-tag (pCMV-FLAG), and the cDNA encoding SERCA2b was subcloned into the pCMV vector with an N-terminal 3xHA-tag (pCMV-3xHA). A cDNA encoding the N-terminal region of TER (1–81aa, N-term), the TER mutant lacking the N-terminal region (82–308aa, ΔN) or the TER mutant lacking the C-terminal region (1–278aa, ΔC) was subcloned into the pCMV–FLAG vector. To generate an siRNA-resistant TER construct [FLAG–TER (rescue)], six silent mutations were introduced by site-directed mutagenesis in the siTER #1 target sequence in the pCMV–FLAG–TER. The cDNA encoding human NFAT4 (1–407aa) was subcloned into pCA vector with a C-terminal EGFP-tag. A plasmid encoding G-CEPIA1*er* (20) was a kind gift from Dr Masamitsu Iino (Nihon University School of Medicine). For expressing recombinant proteins in *E. coli*, the cDNA encoding the N-terminal region of TER or a cDNA encoding the C-terminal region of TER (279–308aa, C-term) was subcloned into the pGEX-4T-1 vector (GE Healthcare). For generating recombinant baculoviruses, the cDNA encoding TER was subcloned into the pFastBacFLAGa vector which was generated by the replacement of the N-terminal 6 × His-tag in the pFastBacHTa vector (Invitrogen) with a FLAG-tag. The cDNA encoding SERCA2b was subcloned into the pFastBac1 vector (Invitrogen) with an N-terminal PA-tag.

### Reagents

The following reagents were purchased from the indicated manufacturers: mouse anti-SERCA2b mAb (clone IID8 (61), Cat. No. sc-53010, for Western blotting) from Santa Cruz Biotechnology; mouse anti-SERCA2b mAb (clone 2A7-A1 (62), Cat. No. NB300-581, for immunostaining) from Novus; rabbit anti-TER polyclonal antibody (pAb) (Cat. No. A305-515A) from Bethyl Laboratories; horseradish peroxidase–conjugated Strep-Tactin from IBA Lifesciences; rat anti-HA mAb (clone 3F10, Cat. No. 11867431001) from Roche; mouse anti-HA mAb (clone 16B12, Cat. No. 901513) from BioLegend; rabbit anti-FLAG pAb (Cat. No. F7425), mouse anti-FLAG mAb (clone M2, Cat. No. F1804), mouse anti-β-actin mAb (clone AC-74, Cat. No. A2228) from Sigma; rabbit anti-Calnexin mAb (clone C5C9, Cat. No. 2679S) and normal rabbit IgG (Cat. No. 2729S) from Cell Signaling Technology.

### Cell lines

HEK293 cells were maintained in Dulbecco's modified Eagle's medium (DMEM)/F-12 supplemented with 5% fetal bovine serum, penicillin, and streptomycin. The HEK293 cell clones stably expressing Strep-TER were established as described previously (63). HuH-7 cells were maintained in DMEM supplemented with 10% fetal bovine serum, 1% nonessential amino acids (Gibco), penicillin, and streptomycin. Primary human keratinocytes (adult pooled, Cat. No. C-12006, PromoCell) were maintained in Keratinocytes Growth Medium 2 (PromoCell) according to the manufacturer’s instructions.

### Purification of the TER-binding proteins

Cells stably expressing Strep-TER from four 10-cm dishes were lysed by sonication in buffer A [20-mM Tris HCl (pH 8.0), 150-mM NaCl, 1-mM DTT, 2-mM MgCl_2_, and 1-mM CaCl_2_] with a protease inhibitor cocktail. Solubilization was performed by adding Triton X-100 to a final concentration of 1% and subsequent agitation at 4 °C for 30 min. The lysates were then centrifuged at 100,000*g* for 30 min, and the resultant supernatant was applied to a column with 100 μl of Strep-Tactin Sepharose (IBA Lifesciences). After being washed extensively with buffer A with 1% Triton X-100, bound proteins were eluted with buffer B [118-mM Tris HCl (pH 8.0), 150-mM NaCl, 1-mM EDTA, 11-mM desthiobiotin]. The eluted samples were subjected to SDS-PAGE followed by sliver staining with Silver Stain MS kit (Wako) or Western blotting. The bands of interest in the silver-stained gel were cut out and subjected to mass spectrometry analysis.

To examine the effect of Tg on the binding of Strep-TER and SERCA2, cells were treated with 1-μM Tg (Sigma) or dimethyl sulfoxide (DMSO) for 90 min. The cells were then lysed by sonication in buffer A with protease inhibitor cocktail and phosphatase inhibitor cocktails 2 and 3 (Sigma). The cell lysates were solubilized and centrifuged as described above. The resultant supernatant was incubated with Strep-Tactin Sepharose at 4 °C for 2 h. The Strep-TER complex was extensively washed with buffer A with 1% Triton X-100 and eluted with the SDS sample buffer. The obtained samples were subjected to SDS-PAGE and Western blotting.

### Mass spectrometry

The samples were subjected to in-gel digestion with 10 μg/ml sequencing grade modified trypsin (Promega) overnight at 37 °C (64). The digested peptides were eluted with 0.1% formic acid and were subjected to LC-MS/MS analysis, which was performed on a LCMS-IT-TOF (Shimadzu) interfaced with a nano reversed-phase liquid chromatography system (Shimadzu). LC separation was performed using a PicoFrit BetaBasic C18 column (New Objective) at 300 nl/min. Peptides were eluted using gradients of 5 to 40% acetonitrile in 0.1% formic acid and sprayed directly into the mass spectrometer. The heated capillary temperature and electrospray voltage were set at 200 °C and 2.5 kV, respectively. MS/MS data were acquired in the data-dependent mode by LCMS solution software (Shimadzu). The mass spectrometry search parameters were as follows: peak list-generating software, Mascot Distiller (version 2.4.3.3, Matrixscience); search engine, Mascot (version 2.3.2, Matrixscience); database, SwissProt 2017_05 (taxonomy, all); the number of entries in the database, 554,515 sequences; specificity of the protease used to generate peptides, trypsin (the C-terminal side of K or R unless the next residue is P); the number of missed and/or nonspecific cleavages permitted, one missed cleavage; fixed modifications, none; variable modifications, carbamidomethyl (C) and oxidation (M); mass tolerance for precursor ions, ± 0.05 Da; and mass tolerance for fragment ions, ± 0.05 Da. The criteria for protein identification were as follows: (1) Mascot scores above the statistically significant threshold (*p* < 0.05) and (2) at least one top-ranked unique peptide matching the identified protein.

### Coimmunoprecipitation

For the immunoprecipitation of endogenous TER, cells were lysed by sonication in buffer C [20-mM Tris HCl (pH 7.5), 150-mM NaCl, 2-mM MgCl_2_, and 1-mM CaCl_2_] with protease inhibitor cocktail. The cell lysates were solubilized and centrifuged as described above, except that 1% DDM (Dojindo) was used to solubilize the lysates of primary keratinocytes or HuH-7 cells. The resultant supernatant was incubated with normal rabbit IgG or rabbit anti-TER pAb at 4 °C for 16 h. The immunocomplex was then precipitated with Protein G Sepharose (GE healthcare) at 4 °C for 1 h, extensively washed with buffer C with 1% Triton X-100 or DDM, and eluted with the SDS sample buffer. For the immunoprecipitation of FLAG-tagged protein, the indicated combination of plasmids was transfected into HEK293 cells using polyethylenimine (Polysciences). After 24 h, cell lysates were prepared and subjected to immunoprecipitation as described above with the exception that the supernatant was incubated with mouse anti-FLAG mAb at 4 °C for 2 h.

### Immunofluorescence microscopy

Cells were grown on coverslips coated with poly-L-lysine and then fixed with 4% paraformaldehyde in phosphate buffer (Wako). The fixed cells were permeabilized with 100 μg/ml digitonin in PBS for 15 min at room temperature (RT), and then blocked with 1% bovine serum albumin in PBS for 1 h at RT. The samples were incubated with primary antibodies diluted in Can Get Signal immunostain B (TOYOBO) at 4 °C for 16 h, and then with secondary antibodies conjugated with Alexa Fluor 488 or 568 (Invitrogen) diluted in a blocking buffer at RT for 30 min. After being washed with PBS, the samples were mounted and examined using a laser-scanning microscope LSM510 META (Carl Zeiss) with a 63 × oil-immersion lens. Collected data were saved as 8-bit TIFF files and processed using Adobe Photoshop software. Colocalization of TER and SERCA2b was quantified by determining Pearson’s correlation coefficients between the two channels using Coloc 2 plug-in in Fiji (65).

### NFAT4-GFP imaging and analyses

At 48 h after siRNA transfection, HEK293 cells were transfected with NFAT4-GFP by using Effectene (Qiagen) and further incubated for 24 h. The cells were washed with serum-free DMEM/F-12 containing 2-mM CaCl_2_ and incubated in this media for 4 h. The cells were then treated with 100-μM ATP for 5 min and fixed as described above. The samples were incubated with DAPI (WAKO) diluted in PBS at RT for 10 min, mounted, and examined as described above. To quantify the nuclear translocation of NFAT4-GFP, the nuclear and cellular masks were created for each cell from the images of the DAPI and GFP channels, respectively. Next, the areas and mean GFP fluorescence intensities in these masks were measured after background subtraction, and the total GFP fluorescence intensities were determined by multiplying the areas by the mean fluorescence intensities. The mean GFP fluorescence intensity in the cytosol was then calculated with the following equation: (the total fluorescence intensity in the cellular mask − the total fluorescence intensity in the nuclear mask)/(the area of the cellular mask − the area of the nuclear mask). Finally, the ratio of the mean nuclear GFP fluorescence intensity to the mean cytosolic GFP fluorescence intensity was calculated. We performed these analyses with a custom-made macro in Fiji.

### Recombinant proteins

FLAG–TER or PA-SERCA2b was expressed in Sf21 cells using the Bac-to-Bac baculovirus expression system (Invitrogen) according to the manufacturer’s instructions. Cells expressing FLAG–TER were lysed by sonication in buffer D [50-mM Hepes–NaOH (pH 7.0) and 500-mM NaCl] with protease inhibitor cocktail. The lysates were centrifuged at 2000*g* at 4 °C for 5 min to remove unbroken cells and debris, and then centrifuged at 100,000*g* at 4 °C for 30 min. The resultant pellets were resuspended in buffer D with 1% DDM, 20% (w/v) glycerol, and protease inhibitor cocktail and homogenized by passing through 25-G needles. Solubilization was performed at 4 °C for 16 h with agitation, and the samples were centrifuged at 100,000*g* at 4 °C for 30 min. The resultant supernatants were incubated with anti-FLAG M2 affinity gel (Sigma) at 4 °C for 2 h. FLAG–TER immobilized on the resin was extensively washed with buffer D with 1% DDM and 20% (w/v) glycerol and then with buffer E [50-mM Hepes–NaOH (pH 7.0), 100-mM NaCl, 1% DDM, and 20% (w/v) glycerol]. Cells expressing PA-SERCA2b were lysed by sonication in buffer F [50-mM Hepes–NaOH (pH 7.0) and 100-mM NaCl, 1-mM MgCl_2_, 1-mM CaCl_2_, and 20% (w/v) glycerol] with protease inhibitor cocktail. Solubilization was carried out by adding DDM to a final concentration of 1% and subsequent agitation at 4 °C for 16 h. The samples were then centrifuged at 100,000*g* at 4 °C for 30 min. The resultant supernatants were incubated with anti-PA-tag NZ-1 Sepharose (WAKO) at 4 °C for 2 h. The resin was washed with buffer F with 1% DDM, with 0.3% DDM, and then with 0.1% DDM. The elution was performed with buffer F with 0.1% DDM and 200 μg/ml PA-tag peptide (WAKO).

GST, GST-TER-N-term, and GST-TER-C-term were expressed in *E. coli* BL21 (DE3). Cells were then lysed by sonication in buffer G [20-mM Tris HCl (pH 7.5), 150-mM NaCl, 1-mM DTT] with 1-mM EDTA and protease inhibitor cocktail. Solubilization was performed by adding Triton X-100 to a final concentration of 1% and subsequent agitation at 4 °C for 30 min. The samples were then centrifuged at 100,000*g* at 4 °C for 30 min, and the resultant supernatants were incubated with glutathione Sepharose (GE Healthcare) at 4 °C for 1 h. Each GST-tagged protein on the resin was extensively washed with buffer G with 1% Triton X-100 and then with buffer C with 1-mM DTT and 1% Triton X-100.

### Binding assays

The binding experiment between PA-SERCA2b and FLAG–TER was performed in buffer H [50-mM Hepes–NaOH (pH 7.0), 100-mM NaCl, 1-mM MgCl_2_, 5-mM EGTA, 1% DDM, 20% (w/v) glycerol] with or without 0.77-mM CaCl_2_. The free concentration of Ca^2+^ in buffer H with 0.77-mM CaCl_2_ was 100 nM, which was calculated by the chelator program (66). For Tg treatment, 0.25-μM PA-SERCA2b was treated with 2.5-μM Tg or DMSO at 37 °C for 10 min. PA-SERCA2b (50 pmol) was incubated with the anti-FLAG mAb resin with or without immobilized FLAG–TER (125 pmol) at 4 °C for 2 h. After being extensively washed, the bound proteins were eluted with the SDS sample buffer. The obtained samples were subjected to SDS-PAGE followed by Coomassie Brilliant Blue staining.

The pull-down experiments with GST-tagged proteins were performed in buffer C with 1-mM DTT and 1% Triton X-100. Plasmid transfection into HEK293 cells and cell lysate preparation were performed as in the “Coimmunoprecipitation” section. After ultracentrifugation, the supernatants were incubated with each GST-tagged protein immobilized on 50 μl of the glutathione Sepharose at 4 °C for 2 h. After being extensively washed, the bound proteins were eluted with the SDS sample buffer. The obtained samples were subjected to SDS-PAGE followed by Coomassie Brilliant Blue staining or Western blotting.

### ATPase assay using microsomal fractions

The preparation of microsomal fractions was performed as described previously (67) with modification. Plasmid transfection was performed as in the “Coimmunoprecipitation” section. After 24 h, cells from six 10-cm dishes were swollen in buffer I [10-mM Tris HCl (pH 7.5), 0.5-mM MgCl_2_, 1-mM DTT] with protease inhibitor cocktail on ice for 10 min. The cells were homogenized using a glass homogenizer with a Teflon pestle (10 strokes), and the homogenates were diluted with an equal volume of buffer J [10-mM Tris HCl (pH 7.5), 0.5 M sucrose, 300-mM KCl, 40-μM CaCl_2_] with a protease inhibitor cocktail. The samples were then centrifuged at 1000*g* at 4 °C for 10 min and then at 6000*g* at 4 °C for 10 min. The resultant supernatants were centrifuged at 100,000*g* at 4 °C for 30 min, and the pellets were resuspended with a 1:1 mixture of buffer I and buffer J with protease inhibitor cocktail. The suspensions were centrifuged again at 100,000*g* at 4 °C for 30 min, and the pellets (microsomal fractions) were homogenized in a reaction buffer [50-mM Hepes–NaOH (pH 7.0), 100-mM NaCl, 20% (w/v) glycerol] with 20-μM CaCl_2_ by passing through 25 G needles.

SERCA2b ATPase assay was performed using the EnzChek phosphate assay kit (Invitrogen) as described previously (36, 68) with modification. 30 μg of the microsomal proteins was incubated in the reaction buffer with 50-mM NaN_3_, 5-mM MgCl_2_, 0.37-mM CaCl_2_, 1-mM EGTA (free Ca^2+^ concentration is 320 nM), 1-μM A23187 (Cayman), 200-μM 2-amino-6-mercapto-7-methylpurine ribonucleoside, and 1 U/ml purine nucleoside phosphorylase, supplemented with DMSO or 1-μM Tg at 37 °C for 10 min. The ATPase reaction was then started by adding 1-mM ATP. Inorganic phosphate (P_i_) generated by ATP hydrolysis caused the enzymatic conversion of 2-amino-6-mercapto-7-methylpurine ribonucleoside by purine nucleoside phosphorylase, resulting in a shift of absorbance maximum to 360 nm, which was measured with Novaspec Plus spectrophotometer (GE healthcare). The concentration of released P_i_ was calculated as described previously (68). The SERCA2b-dependent P_i_ release was determined by subtracting the amount of P_i_ released in the presence of Tg from that in the presence of DMSO.

### RNA interference

Silencer select predesigned siRNAs (ID: s18269, TER #1; s228511, TER #2) and Silencer select negative control siRNA #2 were purchased from Thermo. Cells were transfected with each siRNA (10 nM) using LipofectAmine RNAiMAX (Invitrogen) according to the manufacturer’s instructions. After 48 or 72 h, cells were subjected to the subsequent experiments. For the reconstitution of TER expression in TER-depleted cells, siTER #1 (10 nM) and FLAG–TER (rescue) were transfected into HEK293 cells with LipofectAmine 2000 (Invitrogen) according to the manufacturer’s instructions. After 72 h, cells were subjected to the subsequent experiments. To check the knockdown efficiency or the reconstitution of TER expression, the whole-cell lysates prepared by sonication were subjected to SDS-PAGE followed by Western blotting.

### Ca^2+^ measurements

For the indirect measurement of the ER Ca^2+^ content, cells were loaded with 5-μM Fluo 4-AM (Dojindo) at 37 °C for 1 h and then suspended in Ca^2+^-free Hank’s balanced salt solution (HBSS) [HBSS without Ca^2+^ and Mg^2+^ (Gibco) supplemented with 10-mM Hepes–NaOH (pH 7.5), 1-mM MgCl_2_, and 0.5-mM EGTA]. The cells were transferred to a 96-well plate, and the fluorescence values were measured using Fluoroskan Ascent FL microplate reader (Thermo) at 37 °C with 485-nm exitation and 538-nm emission. After the baseline fluorescence values were obtained, 1-μM Tg was added to each well. The fluorescence values were then measured every 5 s for a period of 10 min. The data in each independent experiment were the average value from 6 wells per condition and expressed as the change in fluorescence values relative to baseline (ΔF/F_0_).

The direct measurement of the ER Ca^2+^ levels was carried out with G-CEPIA1*er* (20). At 48 h after siRNA transfection, HEK293 cells were transfected with G-CEPIA1*er* by using Effectene and further incubated for 24 h. The cells were then suspended in Ca^2+^-free HBSS and transferred to a 96-well plate. The fluorescence values were measured at 37 °C as described above. After the baseline fluorescence values were obtained, 1-μM Tg was added to each well. The fluorescence values were then measured every 10 s for a period of 15 min. The data in each independent experiment were the average value from 2 wells per condition and expressed as the fluorescence values relative to the final fluorescence value (F/F_final_).

For the measurement of the cytosolic Ca^2+^ responses, cells were plated on a 96-well plate coated with poly-L-lysine 16 h before subsequent experiments. The cells were loaded with Fluo-4 as described above and then incubated in HBSS containing Ca^2+^ and Mg^2+^ (Gibco) supplemented with 10-mM Hepes–NaOH (pH 7.5) for >30 min or in Ca^2+^-free HBSS for 5 min at 25 °C while measuring the fluorescence values. After the baseline fluorescence values were obtained, 100-μM ATP with or without 1-μM Tg was added to each well. The fluorescence values were then measured every 2 s for the periods indicated in the figures. The data in each independent experiment were the average values from 2 or 3 wells per condition and expressed as the change in fluorescence values relative to baseline (ΔF/F_0_) or the percentage of the maximum value in each trace. To determine decay time constants, the data were fitted to a single exponential function: ΔF/F_0_ = ΔF/F_0, peak_ × e^−t/τ^, where t and τ represent the time after the peak and the decay time constant, respectively. The data from the peak until 100 s (for ATP) or 300 s (for ATP and Tg) later were analyzed with Microsoft Excel software.

## Data availability

Raw mass spectrometry data were deposited in PRIDE (Project ID: PXD020792) and can be accessed at https://www.ebi.ac.uk/pride/archive/projects/PXD020792. All remaining data are contained within the article.

## Conflict of interest

The authors declare no conflicts of interest in regards to this article.
