# Supplementary material for: Trans-2-enoyl-CoA reductase limits Ca2+ accumulation in the endoplasmic reticulum by inhibiting the Ca2+ pump SERCA2b
Source: J Biol Chem. 2021 Jan 19;296:100310. doi: 10.1016/j.jbc.2021.100310 (PMC7949109; doi:10.1016/j.jbc.2021.100310)
Supplement: Supplemental Figures S1–S2 and Table S1 [file mmc1.pdf]

Supporting information

Trans-2-enoyl-CoA reductase limits Ca^{2+} accumulation in the endoplasmic reticulum by inhibiting SERCA2b Ca^{2+} pump

Yasunori Uchida, Yasunori Yamamoto, Toshiaki Sakisaka*

Division of Membrane Dynamics, Department of Physiology and Cell Biology, Kobe University School of Medicine, Kobe 650-0017, Japan

*Corresponding author: Toshiaki Sakisaka

E-mail: sakisaka@med.kobe-u.ac.jp; Tel. 81-78-382-5727; Fax. 81-78-382-5419.

Materials included:

- Supplementary Table S1
- Supplementary Figure S1, S2

Table S1. Mass spectrometry data for the identification of Strep-TER binding proteins.

Band	Accession # (SwissProt)	# of peptides assigned	% coverage
p400	PRKDC_HUMAN	34	8
p180	NU205_HUMAN	35	17
p170	MON2_HUMAN	19	11
p170	AT2A2_HUMAN	16	16
p95	AT2A2_HUMAN	6	6
p55	TBB5_HUMAN	11	32

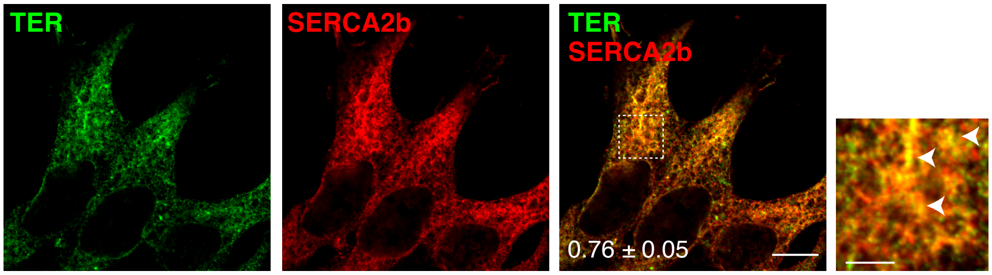

Figure S1. Tg does not affect the co-localization between TER and SERCA2b. HEK293 cells were treated with 1 µM Tg for 90 min, fixed and immunostained with anti-TER pAb and anti-SERCA2b mAb. Data are representative of 2 independent experiments. The magnified view of the boxed area is shown in the right panel. Arrowheads indicate the regions where TER and SERCA2b are co-localized. (Scale bar, 10 µm in the merged image and 3 µm in the magnified image). Pearson's coefficient between TER and SERCA2b is indicated in the merged image (mean ± S.D., n = 26 cells).

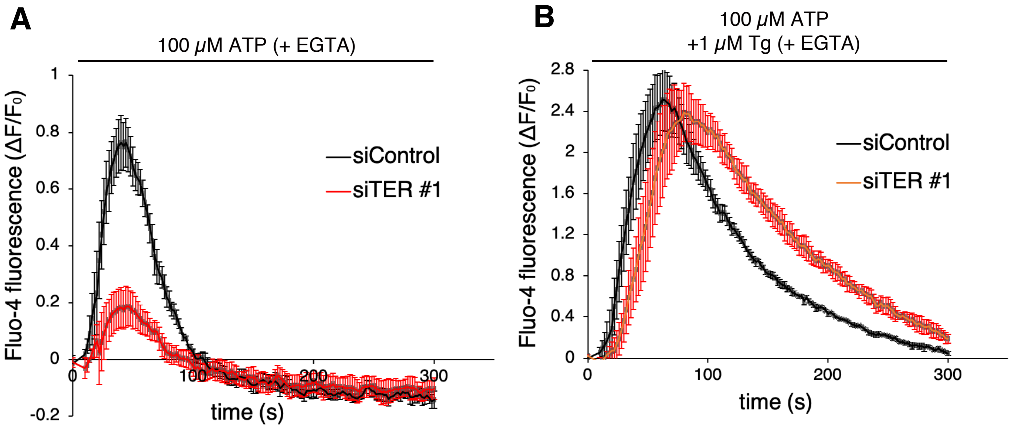

Figure S2. TER depletion attenuates cytosolic Ca^{2+} responses following ATP application in the absence of extracellular Ca^{2+}. **A**. Ca^{2+} responses elicited by ATP. siControl or siTER #1 was transfected into HEK293 cells. After 48 h, the cells were loaded with Fluo-4, and then incubated in Ca^{2+}-free buffer supplemented with 0.5 mM EGTA. After 5 min, 100 μM ATP was added (time = 0 s), and the Fluo-4 fluorescence was measured every 2 s for 5 min. The changes in fluorescence values relative to baseline ($\Delta F/F_0$) were plotted as a function of time. Data represent mean ± S.E.M. from 4 independent experiments. **B.** Ca^{2+} responses elicited by ATP in the presence of Tg. Cells were treated as in (A), except that ATP was added with 1 μM Tg. Data represent mean ± S.E.M. from 3 independent experiments.
